# Special Issue: “Computational Mechanics of Structures and Materials”

**DOI:** 10.3390/ma16165617

**Published:** 2023-08-14

**Authors:** Michele Bacciocchi, Angelo Marcello Tarantino, Raimondo Luciano, Carmelo Majorana

**Affiliations:** 1 Department of Economics, Science and Law, University of San Marino, Via Consiglio dei Sessanta 99, 47891 Dogana, San Marino; 2 DIEF—Department of Engineering “Enzo Ferrari”, University of Modena and Reggio Emilia, Via P. Vivarelli 10, 41125 Modena, Italy; angelomarcello.tarantino@unimore.it; 3 Engineering Department, University of Napoli Parthenope, Via Ammiraglio Ferdinando Acton, 80133 Napoli, Italy; raimondo.luciano@uniparthenope.it; 4 Department of Civil, Environmental and Architectural Engineering (DICEA), University of Padova, Via F. Marzolo, 35131 Padova, Italy; carmelo.maiorana@unipd.it

Computational methods have always affected many engineering fields due to their enormous potential and ability to facilitate various tasks. This statement is surely true as far as the research in structures and materials is concerned. In fact, it can be observed that the body of literature focused on computational and numerical methods for solving various structural problems and characterizing different constituents and materials is huge. This proves not only the attractiveness of this broad topic, but also its potential in developing further advancements in these contexts.

For these reasons, the Guest Editors decided to organize a Special Issue focused on “Computational Mechanics of Structures and Materials”, to collect innovative investigations dealing with accurate, reliable, and effective numerical approaches in the field of both structural mechanics and mechanics of materials. To this aim, a broad scope has been defined, accepting not only finite element or finite-element-based methods, but also different computational techniques involving the achievement of solutions characterized by higher accuracy and reliability. The innovation demonstrated in dealing with advanced materials and constituents is a positive feature of all submitted papers that contributed to their acceptance.

Over several months, this Special Issues constantly proved to be a success, attracting and collecting many interesting submissions. The first heartfelt thanks is directed to all the authors from many different countries of the world who decided to contribute to the collection. Specifically, nineteen papers (out of the twenty-six submitted) have been published, passing through a meticulous review process. Sincere gratitude has to be expressed to the experts who reviewed the papers in spite of their many personal and academic responsibilities.

This great achievement has been made possible thanks to the constant support provided by Ms. Cecilia Zhang, the Section Managing Editor. The Guest Editors would like to thank her for the commitment given to the Special Issue. Finally, the whole editorial team of *Materials*, including the Editors-in-Chief, must be mentioned. They made the management of this Special Issue possible.

To celebrate the success of this commitment, a brief review of the works included in the collection is presented, highlighting the main advancements obtained in the field of computational mechanics.

Gawryluk presented a discussion on the appropriate choice of boundary conditions in structures subjected to a failure analysis. In particular, the research is focused on a thin-walled laminated angle column under compression. The results of both experimental and numerical tests are presented and compared, taking into account different damage criteria [1].

Klimczak and Cecot developed an innovative multiscale finite element method. Their numerical approach proved to be a fast and flexible technique suitable for dealing with heterogeneous materials. The results were obtained in the context of the steady-state heat transfer problem [2].

Bogdan and Radosław employed the finite element method (FEM), including the Johnson–Cook model and the failure parameters of a peculiar class of steel, to simulate the resistance of structures to collisions, shelling, and the impact of pressure waves caused by explosions in water and air in relation to submarines [3].

By means of the finite element method, Wang et al. proposed an investigation on the bearing capacity of high-strength steel-reinforced concrete composite columns. In particular, their analysis emphasized the effect of the confinement of stirrups and steel, highlighting the influence of several parameters and discussing the role of different regulations [4].

Lee and Han studied an infinite isotropic solid embedding different kinds of isotropic and anisotropic spheroidal inclusions. To this aim, they introduced the volume integral equation method (VIEM), which has been demonstrated to be a versatile numerical approach for the three-dimensional elastostatic inclusion problem [5].

Mucha et al. proposed a numerical method to describe a propagating instability phenomenon that effects metals, known as the Portevin–Le Chatelier (PLC) effect. They performed several studies varying the model parameters, which was then efficaciously compared with experimental results [6].

Shi et al. presented a numerical investigation to discuss the effects of the direction and scale of microstructures on the tension problem of a composite plate with a circular hole, proving that these features in solids also have a significant influence on the development of advanced materials. In this context, a micropolar continuum (Cosserat) model was considered [7].

Badea et al. highlighted the limitations of usual finite element models in dealing with tubular structures. By means of a previously developed beam T-junction model, their results provided some strategies to improve the accuracy of beam-element-type approaches, taking into account the real junction stiffness [8].

The paper by Sokołowski and Kamiński is focused on the problem of the topological optimization of corroding structures with uncertainties. They proposed a framework for the reliability-based design optimization (RBDO) of structural elements, considering a corrugated web I-girder as an example. Several numerical approaches have been compared in this context [9].

The work by Bochenek and Tajs-Zielińska also deals with topology optimization. They proposed the concept of the original heuristic topology generator, combining an algorithm with a commercial finite element software, characterized by a significant level of versatility [10].

Chan et al. investigated the mechanics of frozen particle fluid systems by means of a microscale simulation approach based on the discrete element method (DEM) and bonded-particle model (BPM) approach. The results provided by their methodology have been proven to be in good agreement with experimental results [11].

Alrayes et al. developed a numerical approach for simulating crack growth. To this aim, they used a scaled boundary finite element model (SBFEM), emphasizing the importance of simulating the fracture process zone when attempting to describe the cracking behavior of heterogeneous and quasi-brittle materials, taking concrete under monotonic and cyclic actions as an example [12].

Li et al. analyzed the behavior of a hydraulically expanded joint between a tubesheet and titanium tube by means of the finite element method. Their results highlighted the influence of several phenomena with many practical consequences [13].

Alrayes et al. studied mixed-mode crack propagation in concrete through some numerical simulations. They used the scaled boundary finite element method (SBFEM) to assess this phenomenon [14].

Kim et al. developed a nonlinear framework based on the modified couple stress theory to study the axisymmetric bending of circular and annular microplates subjected to thermal and mechanical loads. Their results, obtained via the finite element method, highlighted the effects of several parameters on the bending response [15].

Zhang et al. investigated creep at room temperature by means of a mechanical double-spring steering-gear load table. Their numerical results were successfully compared to experimental tests [16].

Andreotti et al. proposed a methodology to compute the resultant force of ballistic impacts resulting in a full fragmentation of the impactor with no penetration of the target. They discussed the accuracy of different discretization strategies for the corresponding finite element analysis. The results provide many useful suggestions to deal with this kind of problem [17].

The paper by Tahani et al. presents an investigation on the overall mechanical properties of ceramic–metal composites. This was achieved using a computational approach based on the molecular dynamics method. Their results highlighted the influence of several parameters [18].

Finally, the work by Giunta et al. deals with the free vibration analysis of variable-stiffness composite plates. They expanded Carrera’s unified formulation (CUF) plate finite elements to composite laminates reinforced by curvilinear fibers. The effectiveness of the solution was proven through a comparison with results available in the literature or obtained through other commercial codes [19].

The Guest Editors would like to congratulate all the authors for the remarkable achievements illustrated in these papers.
